# Neutrophil-Lymphocyte, Platelet-Neutrophil, and Platelet-Lymphocyte Ratios as Indicators of Sickle Cell Anaemia Severity

**DOI:** 10.4314/ejhs.v33i5.12

**Published:** 2023-09

**Authors:** Chilota Chibuife Efobi, Chisom Adaobi Nri-Ezedi, Chika Sandra Madu, Chuka Charles Ikediashi, Obiora Ejiofor, Chika Ifeoma Ofiaeli

**Affiliations:** 1 Department of Haematology, College of Health Sciences, Nnamdi Azikiwe University, Awka, Anambra State, Nigeria; 2 Department of Paediatrics, Faculty of Medicine, Nnamdi Azikiwe University, Awka, Anambra State, Nigeria; Department of Anatomical Pathology, Federal Medical Center, Umuahia, Abia State, Nigeria; Department of Public Health, Glasgow Caledonian University, Glasgow, Scotland, United Kingdom; Department of Paediatrics, College of Medicine, Chukwuemeka Odumegwu Ojukwu University, Awka, Anambra State, Nigeria; Department of Family Medicine, Faculty of Medicine, Nnamdi Azikiwe University, Awka, Anambra State, Nigeria

**Keywords:** Sickle Cell Anaemia, Disease severity, Neutrophil-Lymphocyte Ratio, Platelet-Neutrophil Ratio, Platelet-Lymphocyte Ratio, Adiposity, Inflammatory markers

## Abstract

**Background:**

Sickle cell anaemia (SCA) imposes a substantial healthcare burden, affecting millions of people worldwide. Understanding the determinants influencing SCA severity is crucial for enhanced disease management and optimized patient outcomes. This study aimed to investigate the relationship between Neutrophil-Lymphocyte Ratio (NLR), Platelet-Neutrophil Ratio (PNR), Platelet-Lymphocyte Ratio (PLR), and SCA severity.

**Methods:**

A cohort of 45 children diagnosed with SCA and undergoing treatment at Chukwuemeka Odumegwu Ojukwu University Teaching Hospital, Awka, was included in this study. Demographic and clinical data, along with laboratory measurements of the aforementioned ratios, were collected. The severity of SCA was assessed using numerical scoring.

**Results:**

The analysis revealed that PNR and PLR emerged as significant predictors of SCA severity, irrespective of the level of adiposity. In contrast, NLR demonstrated no predictive value in relation to SCA severity.

**Conclusion:**

The findings challenge the conventional notion that neutrophils alone play a central role in the pathogenesis of sickle cell crises. These results contribute to a deeper understanding of the disease and provide insights into possible alternative mechanisms underlying SCA severity. Further research is warranted to explore the intricate interplay between platelets, neutrophils, lymphocytes, and other biological factors within the context of SCA. Ultimately, this knowledge may pave the way for targeted interventions and improved management strategies for individuals living with SCA.

## Introduction

Sickle cell anaemia (SCA) is a hereditary haemolytic anaemia that arises from a mutation in the beta-globin gene, leading to the synthesis of abnormal sickle haemoglobin. This debilitating condition is marked by recurrent vaso-occlusive episodes and progressive organ impairment, posing significant challenges for affected individuals, especially children in Nigeria (1,2,3). Despite considerable progress in the understanding of the genetic basis of SCA, there remains a substantial scope for investigating its intricate pathogenesis and wide-ranging clinical presentations.

In addition to the widely acknowledged involvement of sickled red blood cells in sickle cell anaemia (SCA), emerging evidence suggests that other constituents of the immune system, specifically neutrophils and platelets, exert influence on the severity of this condition (4-12). Activated neutrophils, recognized as key players in the inflammatory response, adhere to endothelial cells, thereby inducing the occlusion of blood flow and subsequent tissue injury (4-9). Likewise, platelets, traditionally associated with haemostasis, have also been implicated in the pathophysiology of SCA, owing to their interactions with red blood cells and other immune cells (10-12). The existing body of research has consistently established a correlation between elevated neutrophil levels and severe outcomes in SCA. However, there remains a critical necessity to investigate additional biomarkers that could augment our comprehension of SCA severity in a more comprehensive manner. Neutrophil-Lymphocyte Ratio (NLR), Platelet-Lymphocyte Ratio (PLR), and Platelet-Neutrophil Ratio (PNR) have recently gained attention as potential indicators of inflammation and disease prognosis in various conditions. (13-16) These ratios may offer a unique perspective on the intricate interplay between different immune cell populations and their impact on SCA progression.

The primary objective of this research is to evaluate the utility of NLR, PLR, and PNR as promising markers of SCA severity in Nigerian children. By examining these ratios, we seek to uncover valuable insights into the underlying mechanisms that drive SCA disease progression. Understanding the role of these haematological markers in SCA severity not only contributes to our knowledge of disease pathophysiology but also holds significant clinical implications. Accurate risk stratification and early identification of high-risk individuals can enable targeted interventions and personalized treatment approaches. Furthermore, by providing a cost-effective and readily available screening tool, this research has the potential to improve patient management and resource allocation, particularly in resource-limited settings like Nigeria.

Ultimately, this study aims to enhance our understanding of SCA and its complex interplay with immune cell populations. The findings have the potential to revolutionize disease management, optimize clinical decision-making, and improve the overall quality of life for Nigerian children living with SCA.

## Methods

**Study area**: The study was conducted at Chukwuemeka Odumegwu Ojukwu University Teaching Hospital (COOUTH), a state-owned tertiary healthcare institution situated in Awka-South Local Government Area (L.G.A), Anambra State, Nigeria. Awka, the capital territory of Anambra State, is an urban area. COOUTH serves the healthcare needs of residents in Awka, neighbouring towns, and states. Additionally, the teaching hospital plays a crucial role in training medical personnel and is affiliated with Chukwuemeka Odumegwu Ojukwu University.

**Study design**: This was a retrospective study. Patient case notes were retrieved from the medical records department, covering the period from January 2020 to April 2022.

**Inclusion criteria**: Patients aged 4 to 18 years diagnosed with SCA; Patients with available medical records at the Chukwuemeka Odumegwu Ojukwu University Teaching Hospital (COOUTH) during the specified study period (January 2020 to April 2022)

**Exclusion criteria**: Patients with other hemoglobinopathies or concurrent haematological disorders; patients with incomplete or missing medical records; children below the age of four years were excluded from this study due to the known differences in their blood indices compared to older children and adults, which could influence the utility of the sickle cell disease severity score originally developed for adults. By focusing on the older age group, the study aimed to ensure consistency with the established scoring system and provide relevant insights into disease severity in a population more similar to adults.

**Outcome of the study**: Anticipated findings from this study are expected to provide valuable guidance to health care providers in risk stratification of complications among SCA patients, facilitating timely intervention and ultimately reducing both morbidity and mortality rates associated with this condition.

**Data collection**: Relevant data, including demographic variables, weight, full blood count results, history of blood transfusions, hospital admissions, and complications, were extracted from the patients' medical records and recorded in an excel spreadsheet. The severity of SCA was assessed based on the degree of anaemia using haemoglobin (Hb) levels, white blood cell (WBC) count, history of blood transfusion, and presence of complications (17).

**Scoring of sickle cell anaemia severity**: In order to assess the severity of SCA, a scoring system developed by Okocha et al. (17) was used. The scoring system includes the evaluation of anaemia levels, presence of complications, white cell count, and transfusion history. The scores obtained from each criterion help classify the SCA disease severity as mild, moderate, or severe, providing valuable insights for clinical management and treatment decisions.

**Anaemia score:**

- Hb ≥ 10 g/dl → 0
- Hb ≥ 8 g/day < 10 g/dl → 1
- Hb ≥ 6 < 8 g/dl → 2
- Hb ≥ 4 < 6 g/dl → 3
- Hb < 4 g/dl → 4.

**Complications' score**: Each complication was scored 1 except • Nephropathy → 2 • Stroke → 2. White cell count score:

- Count < 9 × 103 → 0
- Count ≥ 9< 11 × 103 → 1
- Count ≥ 11 < 15 × 103 → 2
- Count ≥ 15 × 103 → 3.

**Transfusion score: Life transfusion rate** = Total number of pints of blood/ Age

Transfusion rate was approximated to the nearest whole number.

**Disease severity** was scored as:

- Mild (<3)
- Moderate (3 ≤ 5)
- Severe (>5)

**Data analysis**: Python 3.10.0 was utilized for data cleaning and analysis. Parametric numerical variables were summarized using descriptive statistics such as means and standard deviations, while non-parametric variables were summarized using medians and interquartile ranges. Categorical variables were presented using frequencies and percentages. Prior to comparing two numerical variables, normality and variance assessments were conducted. We evaluated the association between distinct severity categories of SCA (mild, moderate, and severe) and age, weight, haemoglobin levels, white blood cell count, counts of various immune cells, and relevant ratios. Statistical analysis tools, such as analysis of variance (ANOVA) or the Kruskal-Wallis test, were employed depending on the nature and distribution of the variables. Pearson correlation analysis was conducted to examine the relationship between two numerical variables of interest.

Linear regression analysis was utilized to model the association between SCA severity (dependent variable) and independent variables (NLR, PNR, and PLR). This analysis quantified the strength and direction of the relationship and assessed the predictive capability of the markers. Coefficients were estimated for each variable, indicating the magnitude and direction of their impact on SCA severity. Positive coefficients indicated increased severity, while negative coefficients implied the opposite. The analysis also provided statistical measures such as p-values and confidence intervals to assess coefficient significance and precision, evaluating the statistical validity of the associations. A p-value of <0.05 was considered statistically significant in this study.

## Results

The study enrolled 45 subjects diagnosed with SCA, maintaining a nearly equal male-to-female ratio of 0.96:1. Table 1 provides a comprehensive summary of the participants' demographic characteristics, anthropometric measurements, laboratory parameters, and composite SCA severity score, stratified by gender. The mean age of the entire cohort was 9.8 ± 3.9 years, ranging from 4 to 18 years. Although males were slightly older than females, this was not statistically significant (10.35 ± 4.28 years vs. 9.18 ± 3.51 years; p-value = 0.996). A majority of the participants (64.4%) displayed a healthy weight distribution, while 17.8% were classified as underweight, 11.1% as overweight, and 6.7% as obese. No significant differences in weight distribution were identified between genders.

**Table 1:** Demographic, anthropometric and laboratory parameters in all Subjects with sickle cell anaemia stratified by gender

Variable	Total	Male	Female	t-test/	p-value
	*n=45*	*n=23*	*n-22*	chi^2^	
Age	9.78 ± 3.92	10.35 ± 4.28	9.18 ± 3.51	0.996	0.325
Weight (kg)	30.43 ± 9.99	31.72 ± 10.82	29.08 ± 9.08	0.884	0.381
Weight SDS	-0.46 ± 1.4	-0.58 ± 1.26	-0.32 ± 1.55	-0.614	0.543
Weight Distribution					
Underweight	8 (17.8)	4 (17.4)	4 (18.2)		
Healthy weight	29 (64.4)	17 (73.9)	12 (54.5)	4.042	0.257
Overweight	5(11.1)	2 (8.7)	3 (13.6)		
Obese	3 (6.7)	0 (0.0)	3 (13.6)		
Haemoglobin (g/dl)	9.94 ± 4.58	10.44 ± 6.07	9.41 ± 2.19	0.75	0.457
PCV	27.49 ± 7.74	27.17 ± 9.02	27.82 ± 6.32	-0.278	0.782
WBC	13.86 ± 8.15	14.69 ± 7.57	13.0 ± 8.81	0.688	0.495
ANC	6.71 ± 4.37	6.8 ± 4.14	6.62 ± 4.71	0.136	0.893
Lymphocyte	6.79 ± 4.51	7.45 ± 4.52	6.1 ± 4.5	1.008	0.319
Monocytes	3.59 ± 2.42	3.51 ± 1.78	3.68 ± 2.99	-0.233	0.817
Eosinophils	0.22 ± 0.24	0.26 ± 0.25	0.18 ± 0.22	1.2	0.237
Basophils	0.37 ±0.71	0.29 ± 0.4	0.45 ± 0.93	-0.77	0.446
Platelets	272.02 ± 86.6	290.87 ± 87.79	252.32 ± 82.71	1.515	0.137
NLR	1.13 ± 0.79	1.03 ± 0.76	1.23 ± 0.82	-0.847	0.402
PNR	67.17 ± 55.42	71.25 ± 60.88	62.91 ± 50.15	0.501	0.619
PLR	56.85 ± 37.67	52.93 ± 29.79	60.95 ± 44.83	-0.71	0.482

PCV: Packed cell volume; WBC: White blood cell count; ANC: Absolute neutrophil count; PNR: Platelet-neutrophil ratio; PLR: Platelet-lymphocyte ratio; NLR: Neutrophil-lymphocyte ratio

**SCA-related events and severity: Clinical outcomes and associations**: The study documented a cumulative total of 111 SCA-related clinical outcomes among the participants. The most prevalent clinical events included blood transfusion (40.5%), vaso-occlusive crisis (18.9%), haemolytic crisis (7.2%), chronic leg ulcer (7.2%), and osteomyelitis (7.2%). The severity of SCA, as determined by clinical events, white blood cell count, and haemoglobin concentration, revealed that 18 (40%) subjects had mild SCA, 18 (40%) had moderate SCA, and 9 (20%) had severe SCA. Furthermore, the severity of SCA showed significant associations with haemoglobin concentration, packed red cell volume, neutrophils, lymphocytes, eosinophils, neutrophil-lymphocyte ratio (NLR), platelet-neutrophil ratio (PNR), and platelet-lymphocyte ratio (PLR) (Table 2).

**Table 2:** Relationship between SCA severity with demographic indices, anthropometric measures and laboratory indicators

Name	Total *n=45*	Mild *n=18*	Moderate *n=18*	Severe *n=9*	Test	p-Value
**Age**	10.0(7.0-12.0)	8.5(6.25-11.75)	10.0(7.5-13.0)	12.0(9.0-12.0)	1.507	0.471
**Weight (kg)**	28.0(22.0-35.0)	28.0(22.0-32.0)	30.0(25.25-40.5)	30.0(21.0-35.0)	1.116	0.572
**Weight SDS**	-0.46 ± 1.4	-0.18 ± 1.23	-0.44 ± 1.34	-1.03 ± 1.79	1.129	0.333
**Haemoglobin (g/dl)**	9.3(7.3-11.0)	11.0(10.0-12.0)	8.85 (8.0-11.23)	7.3 (6.6-7.3)	15.448	0.0001**
**PCV**	27.0(22.0-33.0)	31.5(27.75-35.25)	26.6 (24.0-32.25)	22.0 (20.0-22.0)	15.75	0.0001**
**WBC**	12.0(9.0-15.0)	8.3(6.93-10.15)	13.25(11.7-15.0)	19.4(16.5-32.0)	26.37	0.0001**
**ANC**	5.5(3.1-9.8)	3.1 (2.3-4.07)	7.7(5.28-10.32)	11.4(8.6-13.6)	22.761	0.0001**
**Lymphocyte**	5.9(3.7-8.2)	4.25 (3.28-5.78)	6.0(4.12-8.0)	10.7(7.87-16.0)	9.418	0.009**
**Monocytes**	3.0 (2.0-4.0)	2.8 (2.0-4.0)	3.0 (2.0-4.0)	3.0(2.0-3.5)	0.035	0.983
**Eosinophils**	0.13(0.02-0.39)	0.02 (0.0-0.1)	0.16(0.09-0.26)	0.51 (0.41-0.72)	19.14	0.0001**
**Basophils**	0.0(0.0-0.5)	0.1 (0.0-0.3)	0.05 (0.0-0.8)	0.0 (0.0-0.0)	2.381	0.304
**Platelets**	250.0(200.0-318.0)	288.0(209.25-342.0)	250.0(187.25-288.5)	300.0(250.0-350.0)	1.831	0.400
**NLR**	0.84 (0.68-1.27)	0.72(0.49-0.83)	1.17(0.85-1.43)	0.9(0.76-1.24)	9.039	0.011**
**PNR**	53.6(23.8-95.6)	99.7(56.15-135.43)	30.0(22.45-55.47)	23.8(18.8-66.14)	15.078	0.001**
**PLR**	45.5(31.7-74.5)	57.55 (46.5-81.4)	41.7(31.25-49.47)	29.4(13.9-55.67)	7.698	0.021**

** Statistically significant p-values; PCV: Packed cell volume; WBC: White blood cell count; ANC: Absolute neutrophil count; PNR: Platelet-neutrophil ratio; PLR: Platelet-lymphocyte ratio; NLR: Neutrophil-lymphocyte ratio

**Figure 1:**
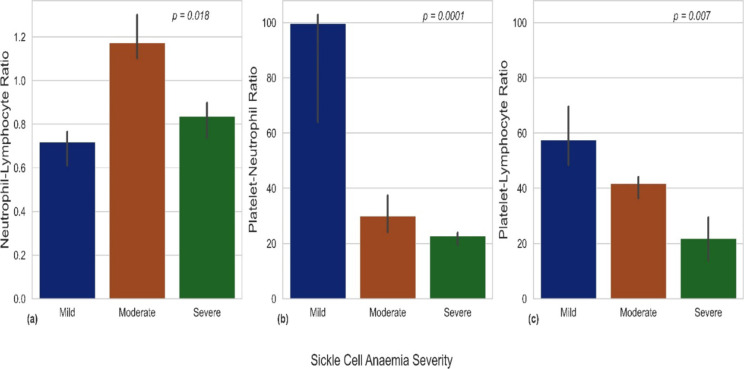
Bar plots of eEstimated medians and standard errors between (a) neutrophil-lymphoctye ratio (b) platelet-neutrophil ratio and (c) platelet-lymphocyte ratio and SCA severity

**Figure 2:**
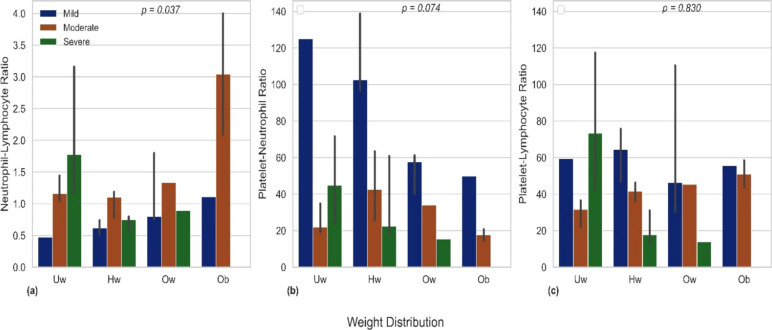
Bar plots of estimated medians and standard errors between (a) neutrophil-lymphoctye ratio (b) platelet-neutrophil ratio and (c) platelet-lymphocyte ratio and weight distribution stratified by SCA severity

**Relationship between NLR, PNR, and PLR with sickle cell anaemia severity**: Table 2 and Figure 1 provide clear evidence that PNR and PLR are elevated in mild cases of sickle cell, with a stronger association observed for PNR. Conversely, NLR does not exhibit a clear association with sickle cell severity. In Figure 2, the association between PNR and sickle cell severity, as depicted in Figure I, remains consistent regardless of adiposity levels.

To further assess the strength of the associations between these ratios and SCA severity, Table 3 presents correlation values. All ratios show statistically significant correlations with numerical SCA severity scores. NLR demonstrates a weak positive correlation (r = 0.366, p-value = 0.014), while PNR and PLR exhibit stronger inverse correlations (r = -0.564, p-value = 0.0001 and r = -0.461, p-value = 0.001, respectively). These findings emphasize the potential of NLR, PNR, and PLR as indicators and predictors of SCA severity.

**Table 3:** Correlation analysis between variables of interest and SCA severity scores

Name	NLR (p-value)	PNR (p-value)	PLR (p-value)	Severity Score (p-value)
**Age**	0.069 (0.652)	0.002 (0.987)	0.021 (0.89)	0.074 (0.627)
**Weight (kg)**	0.068 (0.658)	-0.1 (0.513)	-0.024 (0.873)	0.008 (0.957)
**Weight SDS**	0.001 (0.997)	-0.169 (0.267)	-0.123 (0.42)	-0.214(0.159)
**Haemoglobin**	-0.32 (0.032)**	0.299 (0.046)*	0.176 (0.246)	-0.595 (0.0001)***
**PCV**	-0.31 (0.038)**	0.31 (0.038)*	0.197 (0.196)	-0.532 (0.0001)***
**WBC**	0.283 (0.059)	-0.751 (0.0001)***	-0.733 (0.0001)***	0.814(0.0001)***
**ANC**	0.574 (0.0001)**	-0.895 (0.0001)***	-0.647 (0.0001)***	0.755 (0.0001)***
**Lymphocyte**	-0.249 (0.099)	-0.407 (0.006)**	-0.822 (0.0001)***	0.537 (0.0001)***
**Monocytes**	0.309 (0.039)**	0.114(0.457)	0.397 (0.007)**	-0.035 (0.82)
**Eosinophils**	0.453 (0.002)**	-0.609 (0.0001)***	-0.424 (0.004)**	0.709 (0.0001)***
**Basophils**	0.142 (0.353)	0.165 (0.278)	0.287 (0.056)	-0.171 (0.26)
**Platelets**	-0.35 (0.018)**	0.504 (0.0001)***	0.328 (0.028)*	0.009 (0.954)
**NLR**	1.0	-0.625 (0.0001)***	0.011 (0.945)	0.366 (0.014)*
**PNR**	-0.625 (0.0001)***	1.0	0.703 (0.0001)***	-0.564 (0.0001)***
**PLR**	0.011 (0.945)	0.703 (0.0001)***	1.0	-0.461 (0.001)**
**SCD Score**	0.366 (0.014)*	-0.564 (0.0001)***	-0.461 (0.001)**	1.0

* p<0.05

** p<0.01

*** p<0.001

PCV: Packed cell volume; WBC: White blood cell count; ANC: Absolute neutrophil count; PNR: Platelet-neutrophil ratio; PLR: Platelet-lymphocyte ratio; NLR: Neutrophil-lymphocyte ratio

### Linear regression analysis

**PNR vs. SCA severity**: A linear regression analysis revealed a significant relationship between PNR and SCA severity. The estimated formula indicated that each unit increase in PNR was associated with a decrease of approximately 0.024 units in the predicted SCA severity score (p-value < 0.001, 95% confidence interval: -0.033 to -0.014). This association remained significant even after adjusting for weight, with a decrement of 0.02 units in severity for each unit increase in PNR (p-value < 0.001; CI: -0.028 to -0.011).

**PLR vs. SCA severity**: A linear regression analysis unveiled a significant association between PLR and SCA severity. The obtained estimated equation revealed that each incremental unit in PLR was linked to an approximate reduction of 0.018 units in the projected SCA severity score (p-value < 0.010, 95% confidence interval: -0.031 to -0.005). Notably, this association remained statistically significant even after adjusting for weight, demonstrating a decrease of 0.018 units in disease severity for every unit increase in PNR (p-value < 0.010; 95% confidence interval: -0.032 to -0.004).

**NLR vs. SCA severity**: No significant relationship was observed between NLR and severity.

## Discussion

The primary objective of the current study was to examine the correlation between haematological ratios, namely Neutrophil-to-Lymphocyte Ratio (NLR), Platelet-to-Neutrophil Ratio (PNR), and Platelet-to-Lymphocyte Ratio (PLR), with the severity of sickle cell anaemia (SCA). The findings of this investigation unveiled noteworthy associations between these ratios and the severity of SCA, thereby providing valuable insights into their prospective utility as indicators and prognosticators of disease severity.

The investigation revealed elevated PNR and PLR along with decreased NLR, in mild cases of SCA. This intriguing observation suggests that the interplay between platelets, neutrophils, and lymphocytes may hold a pivotal role in the underlying pathophysiological mechanisms of SCA. Traditionally, platelets have been recognized for their ability to activate neutrophils and release pro-inflammatory mediators, thereby contributing to the progression of sickle cell crises (5,10-12). However, the elevated PNR and PLR observed in mild cases challenge the conventional understanding that increased platelet activation and subsequent interaction with neutrophils contribute to disease severity and inflammation in SCA.

For each incremental unit rise in PNR an PLR, a concomitant statistically significant decrease in the predicted severity score of SCA was observed. These associations remained robust even after adjusting for body weight, reaffirming the independent predictive value of PNR and PLR in assessing the severity of SCA. The findings highlight the potential clinical significance of PNR and PLR as valuable biomarkers for evaluating disease severity in SCA. In contrast, NLR exhibited no discernible predictive value regarding SCA severity, indicating its limited utility as a stand-alone indicator in this context.

Neutrophils have long been recognized for their involvement in the pathophysiology of SCA, particularly in relation to inflammation and endothelial dysfunction. (4-9) However, these current findings suggest that PNR and PLR may provide additional insights into disease severity beyond the sole consideration of neutrophil activity. While the traditional knowledge of neutrophils' role in SCA remains valid, these new findings highlight the potential significance of platelet-related factors in contributing to disease severity. Elevated PNR and PLR and its association with sickle cell severity imply that platelets may play a more substantial role than previously recognized. Therefore, incorporating platelet-related markers, such as PNR and PLR, alongside traditional markers of neutrophil activity, could enhance the understanding and management of SCA. Additionally, the lack of a significant association between NLR and SCA severity further challenges the notion that neutrophils play a central role in the pathogenesis of sickle cell crises. Further research is needed to explore alternative mechanisms and identify additional factors contributing to the severity of SCA.

The implications of this association extend beyond the laboratory, and suggests the involvement of complex biological mechanisms in the pathogenesis of SCA which holds great promise for enhancing our understanding of SCA and its clinical management. By recognizing the impact of these haematological ratios on SCA disease severity, clinicians can gain valuable insights into risk stratification, disease progression monitoring, and treatment response evaluation. Ultimately, this knowledge may pave the way for tailored interventions, improved therapeutic strategies, and better outcomes for individuals worldwide affected by SCA.

It is important to acknowledge the limitations of this study which includes the relatively small sample size. Future investigations incorporating larger cohorts and considering additional clinical and genetic factors would enable the development of more comprehensive predictive models to enhance the understanding and management of sickle cell anaemia severity. Despite its limitations, this study represents the first exploration, to the best of the authors' knowledge, of the association between NLR, PNR and PLR with SCA severity, adding a novel perspective to the existing body of knowledge. The strength of this study lies in its ability to provide clear evidence of elevated PNR and PLR in mild cases of sickle cell disease, indicating their potential as informative markers for disease severity assessment. Additionally, the study highlights the consistent association between PN and PLR with sickle cell severity across different levels of adiposity, underscoring the robustness of this relationship.

In conclusion, this study provides valuable insights into the potential of haematological ratios, namely PNR and PLR, as indicators and predictors of SCA severity. The findings challenge existing paradigms and contribute to a deeper understanding of the complex interplay between haematological components and disease severity in SCA. This knowledge has the potential to improve risk assessment, treatment strategies, and clinical outcomes for individuals worldwide affected by SCA, ultimately making a significant impact on global health.
